# A bibliometric review study of Michael Burawoy

**DOI:** 10.3389/fsoc.2024.1337742

**Published:** 2024-02-14

**Authors:** Aditya Raj, Anushka Sinha

**Affiliations:** Department of Humanities and Social Sciences, Indian Institute of Technology, Patna, India

**Keywords:** bibliometric review, Michael Burawoy, systematic review, public sociology, Global South

## Abstract

This review article utilizes the technique of citation-based bibliometric analysis to provide a comprehensive understanding of the scholarly contributions made by sociologist Michael Burawoy. The most influential academic articles published by Burawoy were retrieved for analyses. Following this, scholars, journals and institutions that he most frequently collaborated with were traced. Further, country-wise analysis of his scholarship was carried out. Lastly, content analyses of retrieved articles identified prominent thematic domains in sociology to which Burawoy contributed, while temporal analyses helped to identify some emerging research hotspots. Findings reveal that historical and cultural context of Burawoy’s research mostly remained confined to the U.S.A., however, he significantly contributed towards the foundation of sociology in the Global South and studied the ensuing global power imbalances. Contemporary sociological thought remains indebted to Burawoy for his comparative study of industrial relations in the 21st century, and recently, his elaboration upon the need for public sociology has taken the discipline in new intellectual directions that appeals to a broader sociological community.

## 1 Introduction

Bibliometric analyses is the application of mathematical and statistical methods to books and other media of communication (Pritchard and Wittig, 1981). Owing to the proliferation of open access databases and availability of free software for analyses, it has become the central means for evaluating scholarly publications and their producers. Although bibliometric studies have been primarily used in natural sciences, recently it has also been used in the broad category of social sciences, especially in disciplines like linguistics (Sun et al., 2021), economics (Samudra, 2023), psychology (Dong et al., 2022), and publication media (Petrovich, 2022). This paper aims to extend this method to the discipline of sociology further by putting the work of American social theorist and ethnographer Michael Burawoy to bibliometric analyses. The academic discipline of sociology has addressed issues of labor, racial justice, globalization and the development of democracy for a long period of time. One salient source of such scholarship is the extensive work of Burawoy. He developed a strong interest in sociology during his undergraduate studies in Cambridge, which was taken up more extensively during his post-graduation at the University of Zambia. After studying the impact of imperialism on independent Africa, he moved to the United States and enrolled as a doctoral student at the University of Chicago. Following his Ph.D., Burawoy went on to become Chair of the department and has also been the president of the American Sociological Association (2004–2006) as well as the International Sociological Association (2010–2014). Influenced by both the Chicago and Manchester schools of sociology, Burawoy noted the shift in Chicago working classes from neighborhood associations to industry based trade unions in the 1920s and 30s (Burawoy, 1982). Later, he explored the shift from despotic to hegemonic control in post-World War II industrial relations and delved into globalization, examining the interplay between global imperialism and the global post-modern (Burawoy et al., 2000a). He has been the recipient of many accolades, including the lifetime achievement award from the American Sociological Association.

## 2 Formulation of the research problem

While Burawoy’s contributions to sociology cannot be denied, it is also true that all quarters of the discipline may not necessarily consider his name when developing a register of prestigious sociologists in the last few decades (Korom, 2020). The direct motivation in this paper for selecting Burawoy for bibliometric analyses was therefore not based on Burawoy’s ubiquitous status as a canonical sociologist of contemporary times. Rather, it was the view that his scholarship has produced debates within the discipline, which continue to find place in indexed publications of influential journals like *American Sociological Review* (Burawoy, 2005a) and *British Journal of Sociology* (Burawoy, 2005b), among others. This point can be illustrated further through his arguments in favor of public sociology, which resonated with many sociologists (Bridger and Alter, 2010; Pathak, 2022), and yet, countless others have been critical of its formulation (Ericson, 2005; Holmwood, 2007). The contentions around Burawoy’s scholarship, for instance, can be seen in the polemical response of Polish sociologist Sztompka, which reflects broader discussions the two scholars had regarding the application of sociological knowledge to various contexts (Sztompka and Burawoy, 2011). Sztompka argued that Burawoy’s framework of public sociology may be well-intentioned and acceptable at the discursive level, but remains deficient in methodological rigor. He wrote that Burawoy’s aspiration for an egalitarian, global sociology may have been misplaced since the very origins of the discipline could be traced to the domain of science, the nature of which is elitist (Sztompka and Burawoy, 2011). These intellectual puzzles and contradictions inherent in Burawoy’s work might therefore be demystified through a fuller assessment of its structure, influence and visibility. The following aims guided the bibliometric analyses:

1. To visualize the network of productive journals and scholars who have published with Michael Burawoy.
2. To reveal the countries which have been globally most influenced by Michael Burawoy’s scholarship and his most significant publications.
3. To map thematically related publications by Michael Burawoy and identify the prominent research clusters.
4. To trace the emerging paths in publications made by Michael Burawoy between 1978 and 2022.

To tackle this objective, a bibliometric approach has been taken. Specifically, full-length, published, peer-reviewed journal articles authored by Burawoy in the English language, between the time-period of 1978 to 2022 has been retrieved from the Elsevier governed electronic database Scopus and analysed. Bibliometric analysis has been used to understand research trends, and this approach can help researchers in sociology to gain better perspective around Burawoy’s legacy. As such, the bibliometric approach employed in this paper has aimed to empirically analyze Burawoy’s most influential research articles, along with the authors and journals he has most frequently collaborated with. It has also tracked the dissemination of Burawoy’s scholarship by looking at the country-wise reception of his work. Further, keywords used frequently by Burawoy in research articles have been identified and content-analysis of term co-occurrence across retrieved articles has been done to provide a broad classification of his most prominent ideas. Thereafter, thematic clusters have been identified, and finally, temporal growth of research themes in the given time period has also been tracked.

## 3 Materials and methods

In this study, the Elsevier governed database Scopus was accessed through institutional credentials of the researchers. There are several strategic advantages to selecting this database. Scopus has a more expansive archive compared to Web of Science (WoS) and can boast of comprising over 23,000 indexed documents (Pranckute, 2021). Scopus allows for the easy exportation of data to multiple software. Other databases or publication platforms such as Sage Publications database had to be excluded from this study because they were not compatible for exportation to the computer software. Further, the search process at Scopus is two-fold, a basic search followed by an advanced search which allows researchers to frame long and complex string of queries with ease. The Scopus database also allows the researcher to look up terms in multiple fields such as title, abstract, keywords, name of the author etc.

The search in this study was conducted by simply including the word “Burawoy” in the author field. Quotation marks were used for ease of interpretation by the database. 178 total documents were retrieved from this initial search after which the documents were filtered based on (a) full name of the author, (b) language, and (c) type of document. Documents authored by anyone other than “Burawoy, M” were excluded from the study at the first stage. Out of the remaining 105 documents, any document that was written in languages other than English was excluded at the second stage. English was chosen because of its international coverage and also because the researchers had the required level of linguistic competency in it. Out of the remaining ninety-five documents, all documents other than full-length research articles were excluded from the search. This was done to create homogeneity in the type of document reviewed and to filter out grey literature such as research blogs, interviews and other modes of informal scholarly communication. Finally, a set of forty-eight research articles were retrieved for full-text review and exported to the computer software for bibliometric analysis. Exported data included growth of publications, languages, countries, journals, citations, funding agencies etc. To analyze the research trends and content analyses, two tools were utilized- (1) Biblioshiny 2.0, which is a graphical web interface from Bibliometrix that runs in the environment of RStudio and (2) VOSviewer 1.16.16, a network visualization tool. Specifically, Scopus database and Biblioshiny 2.0 was used for three-field plot analyses of sources, authors and keywords, country-wise scientific production of the author, and the most cited document of the author. VOSviewer was used for term co-occurrence analyses for thematic clustering and temporal evolution of themes in retrieved literature.

## 4 Results and discussion based on bibliometric analyses

Following are the results of three-field plot analyses of sources, authors and keywords, the country-wise scientific production of the author, and the most cited document of the author. These three indicators initially draw a clear picture of the scholarly impact of Burawoy’s published work on the academic community across the globe.

### 4.1 Three field plot of sources, authors, and keywords

**Figure 1:**
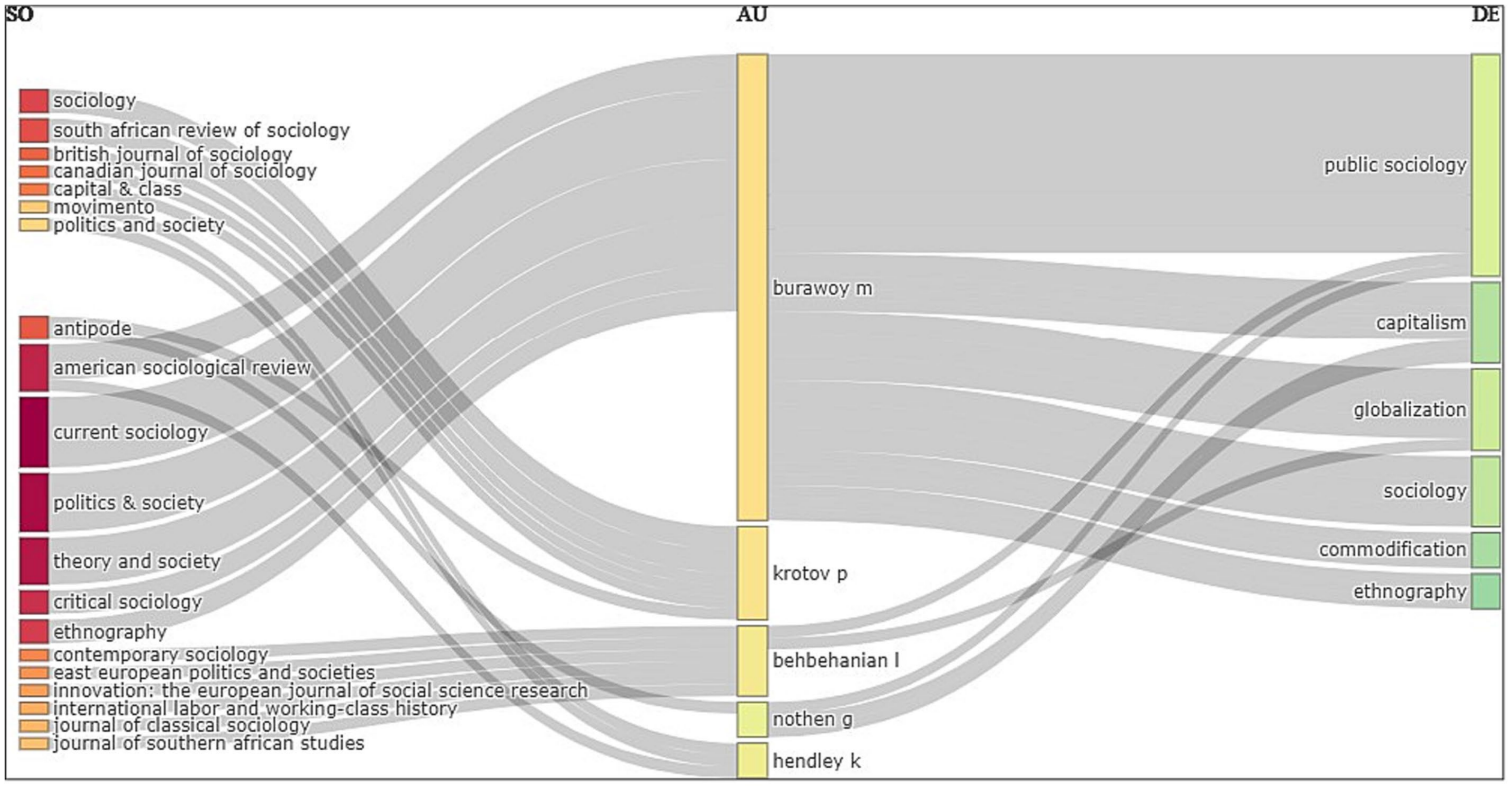
Three-field plot showing the top twenty journals that Michael Burawoy has collaborated with (SO), along with the names of his collaborators (AU) and the corresponding topics that have dominated research trends (DE). Source: Generated by authors from Biblioshiny.

From the visualization in Figure 1, we can note that *Current Sociology*, *Politics and Society*, *American Sociological Review* and *Theory and Society* emerged as the journals that Burawoy has the most number of citations in, while topics such as Public Sociology, Capitalism, and Globalization dominates the research trend. Thereafter, sociologist Pavel Krotov from the University of Wisconsin-Madison and Professor Laleh Behbehanian of University of California, Berkely, emerged as his top collaborators. This result is not surprising given Burawoy’s long-standing professional association with the American Sociological Association, which publishes both the journals *Current Sociology* and *American Sociological Review*. It also shows Burawoy’s early fellowship with Russian sociologist Krotov, who collaborated with him for developing a case study of the debates surrounding Russian trade unions at the time of their transition from state socialism to capitalism (Burawoy and Krotov, 1992). Further, it is reflective of his prominent alliance later with Behbehanian, with whom he constructed an experimental program at University of California, Berkely, titled Global Sociology, Live! The series of seminars, which was later published as an academic paper (Behbehanian and Burawoy, 2012), brought sociologists from across the globe into conversation with each other regarding ways to take the discipline beyond the academy and to the public. Identifying the journals and co-authors with whom Burawoy has published most frequently and locating the dominant keywords may serve as a useful resource for future researchers who may be looking for reference material for applying and extending Michael Burawoy’s scholarship to varied contexts.

### 4.2 Country-wise journal publications

Based on the search conducted on data retrieved from Scopus (Table 1), country-wise origin of journals in which Burawoy has published from 1978 to 2022 has been reflected. Nineteen publications have been recorded by Burawoy in the United States of America, seventeen publications have been produced in United Kingdom, and four publications are to be found in South African region. Further, four publications in Netherlands and one publication each in Canada, Germany, Portugal, and Brazil has been recorded. This is interesting because at first glance, country-wise distribution of Burawoy’s scholarship depicts that there is a high concentration of research in the U.S.A, and U.K. However, it also reflects that due to Burawoy’s post-graduate education in Zambia and subsequent fieldwork on race and class relations in South Africa, the author has a deep and abiding commitment to the development of sociology in the region. These findings indicate the global turn in Burawoy’s scholarship, tracing how he studied the flow of U.S industrial capital from its epicenter to diverse national terrains, including but not limited to South Africa. In the context of India, for example, Burawoy (2013) early research on medium of instruction for university education in the country led to collaborations with sociologists who continue to take his work forward by applying his ideas to socio-political issues such as the migrant workers crisis during the COVID-19 pandemic (Pathak, 2020).

**Table 1:** Country-wise origin of the journals where Michael Burawoy has published between 1978 and 2022.

Sl no.	Country	Number of publications
1.	United States of America	19
2.	United Kingdom	17
3.	South Africa	4
4.	Netherlands	4
5.	Canada	1
6.	Germany	1
7.	Portugal	1
8.	Brazil	1

Generated by authors from Scopus.

### 4.3 Most cited document globally

**Figure 2:**
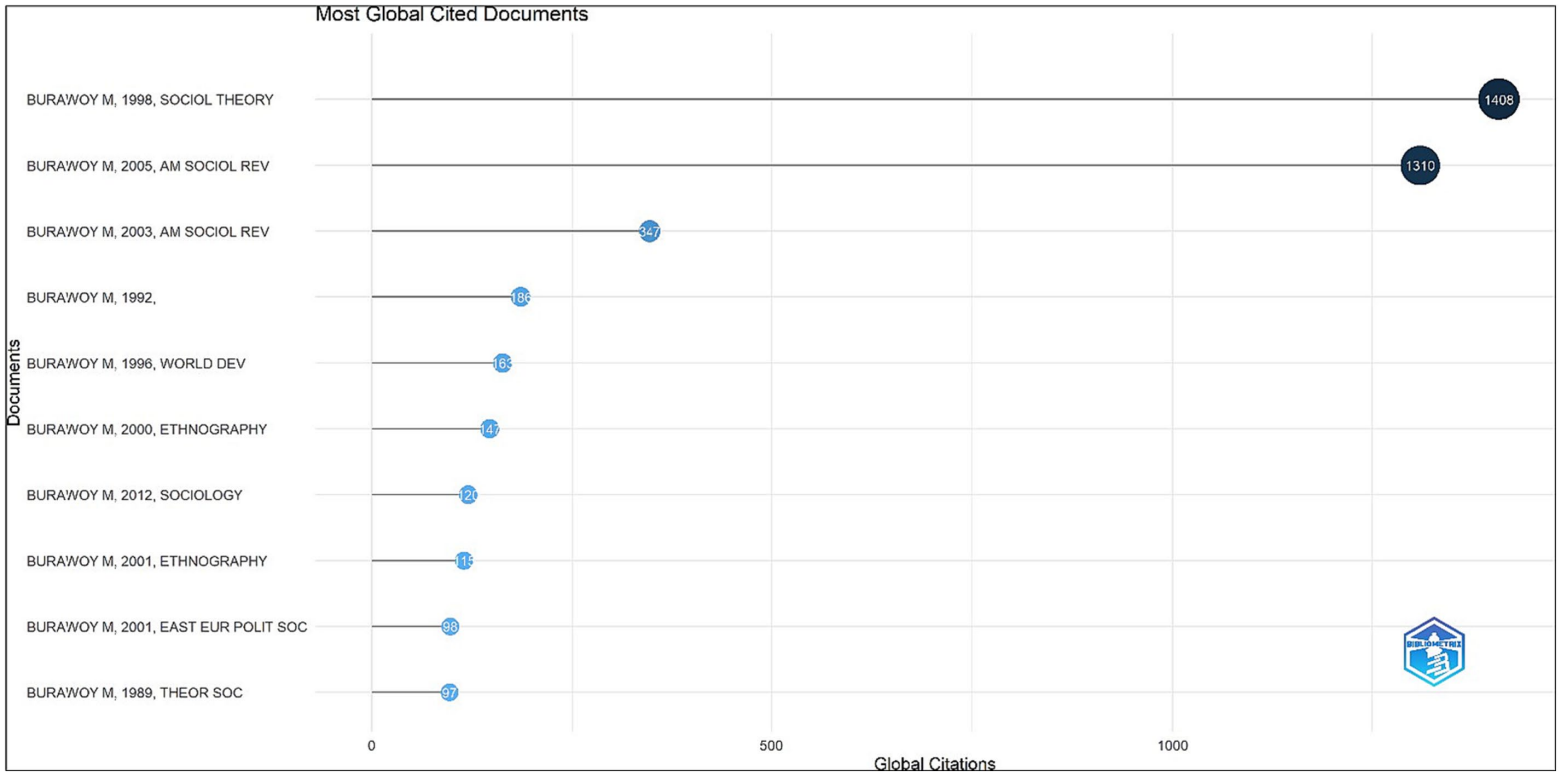
Documents published by Michael Burawoy that are cited most frequently by the academic community. Source: Generated by authors from Biblioshiny.

Figure 2 discusses the top two documents that have been cited the highest number of times by Burawoy. At the top, with 1,408 citations is the paper “The Extended Case Method” (ECM) published in the journal *Social Theory* in 1998. The ECM is a qualitative research method that was originally developed by British socio-cultural anthropologists and later reformulated by Burawoy (1998). It is based on the idea that social actors are not passive recipients of external forces but they actively construct their social reality. According to Burawoy (1998), ECM has three distinct phases-theoretical sensitisation, immersion and induction, and theoretical elaboration. Following this, the second document which has been cited 1,310 times is Burawoy’s presidential speech at the American Sociological Association. This was soon published in the journal *American Sociological Review* where he talked about public sociology being a dialogic reaction between the sociologist and other chosen public(s). Pursuing this line of inquiry further, Burawoy (2005a) reflected on how the discipline can and must break out of the ivory tower to reach the non-academic masses.

## 5 Results and discussion based on thematic clustering and temporal analyses

Retrieved literature was imported to the VOSviewer 1.16.16 software for text mapping and the results have been presented in the following section. Two types of visualizations were carried out with the help of the software. Firstly, network visualization was done on the basis of term co-occurrence to identify the dominant topics that Burawoy has researched on and to depict the relationship between these topics. Secondly, overlay visualization was carried out to provide a timeline of research topic evolution and flag off topics that have been significant to Burawoy in the recent times.

### 5.1 Thematic clustering and analyses

**Figure 3:**
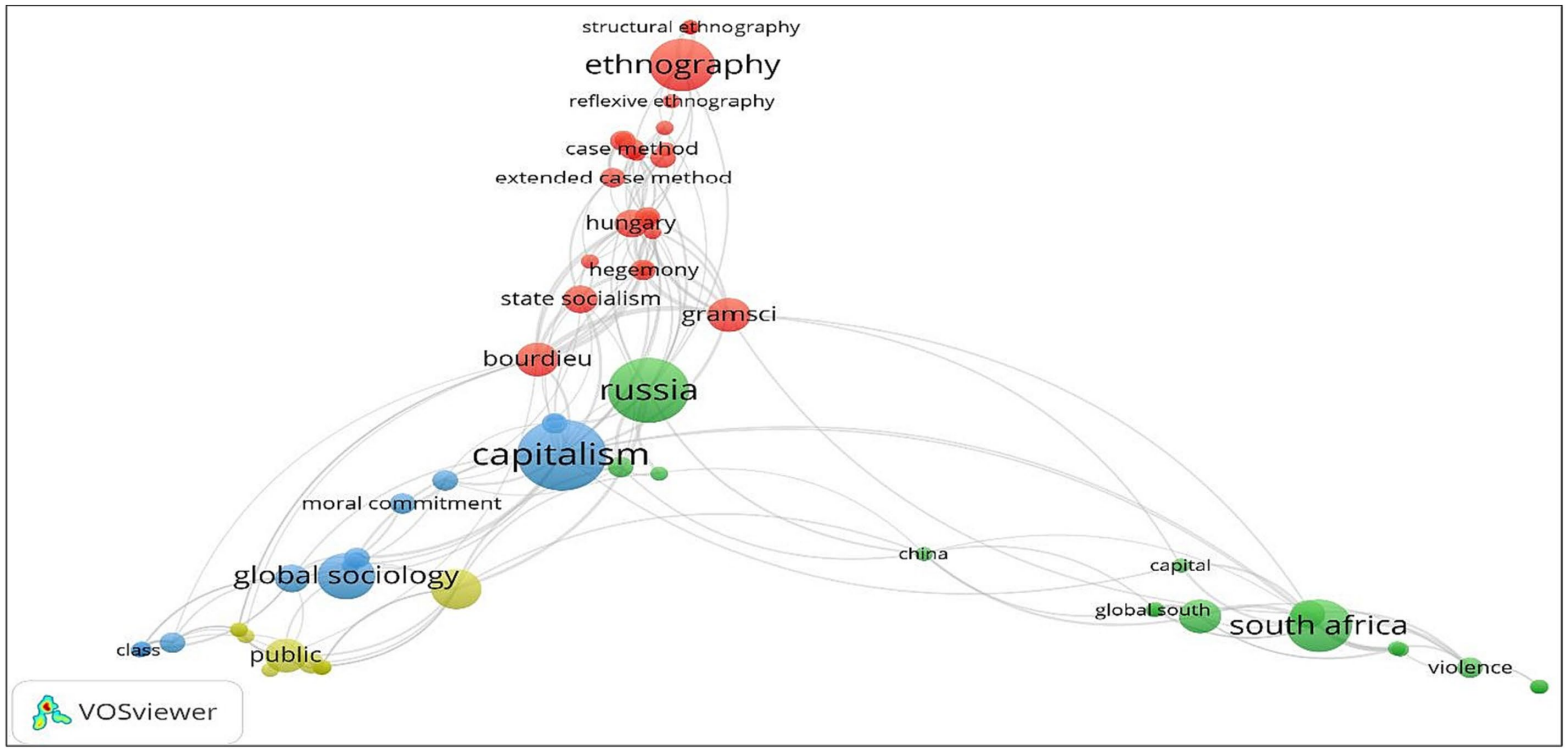
Color-coded thematic clustering of Burawoy’s scholarship based on term co-occurrence in articles. Source: Generated by authors from VOSviewer.

To illustrate the research themes, term co-occurrence for 172 terms was analysed by setting the co-occurrence threshold of terms at two. After this, sixty-three terms or items were brought into visualization. In Figure 3 the size of nodes represent the co-occurences of terms or items. The terms “capitalism”, “ethnography”, “public”, and “South Africa” had the strongest strength. Generally speaking, the distance between two terms or items represent the relative strength of the link between those two terms or items and thus indicate topic similarity. Following this, the links between the selected terms or items was established and signified through a positive numerical value. The higher the numerical value, the stronger the link between terms or items. From Figure 3 it can be deduced that 227 links were established between terms or items. Further, terms or items were categorized into different thematic clusters based on the strength of their links to one another and if terms or items were a part of the same cluster they reflected identical research topics. In Figure 3 each cluster has a different number of terms or items and is displayed by a different color. By further analyzing the largest nodes from each cluster, it is possible to attach thematic labels to the diverse concentrations of Burawoy’s scholarship. The terms or items in Cluster 1(Red) were pertaining to research on the topic of ethnography and related methodological considerations in sociology, specifically the extended case method. As for Cluster 2 (Green), the terms reflected Burawoy’s academic interest in the development of sociology in Global South regions such as South Africa, Russia and China. In Cluster 3 (Blue), the terms were indicative of Burawoy’s Marxist critique of third-wave marketization post the 1980s and his eventual articulation of the need for public discourse in sociology that engaged subjugated subaltern groups. Lastly, in Cluster 4 (Yellow) terms were relating to the classification of four types of sociology propounded by Burawoy and the need for public sociology in particular. Based on co-term analyses, Table 2 provides a more detailed description of the thematic clusters. Based on the four major clusters that have emerged from term co-occurrence analyses (Figure 3), it is possible to identify existing and emerging themes within Burawoy’s scholarship at a glance (Table 2). Each of these themes have been elaborated upon further in this section of the paper.

**Table 2:** List of terms in each thematic cluster based on the diverse concentrations of Michael Burawoy’s scholarship.

Sl no.	Number of terms	Color	Terms
1	22	Red	Bourdieu, class consciousness, comparative analysis, ethnographic research, ethnography, extended case method, Gramsci, hegemony, historical change, Hungary, labor process, positive science, post colonialism, reflexive ethnography, reflexive science, state socialism, structural ethnography, substantialism, symbolic domination, USA, Zambia
2	15	Green	California, capital, China, critical sociology, gobal South, global turn, Harold Wolpe, labor movement, liberation movement, party state, Russia, Russian economy, South Africa, South African sociology, violence
3	15	Blue	Capitalism, case study, class, common sense, economic transition, global sociology, market society, marketization, moral commitment, political practice, public discourse, socialism, subaltern group, third wave marketization, unequal world
4	11	Yellow	Academic field, global field, multiple public, multiple way, pathological form, policy, political field, public, public sociology, sociological ethos, sociological labor

Generated by authors through VOSviewer.

#### 5.1.1 Cluster 1: methodological considerations in sociology

In the first cluster, based on co-occurrence of terms such as “ethnography”, “extended case method”, “reflexivity” etc. a common linkage can be outlined with respect to Burawoy’s contribution towards methodological novelty in sociology. His most prominent contribution in this regard is the Extended Case Method (ECM). It must be noted here that even before Burawoy’s scholarship sought to breathe new life into the ECM, it had been an influential approach in socio-cultural anthropology for decades (Evens and Handelman, 2005; Kempny, 2005). Specifically, the ECM helped anthropologists from Oxford, Cambridge, and Manchester including Evans-Pritchard (1937), Fortes (1949), Gluckman (1955), and Turner et al. (2017) to write evocative details of social life in central and southern Africa. Taking this intellectual tradition forward, Burawoy applied the ECM to critically think through existing theories and assumptions as well as challenge them. South African-British anthropologist Max Gluckman had distinguished Extended Case Method from two more constrained applications of the case study, both of which had a propensity to advance structuralism’s interest in social morphology, i.e., apt illustration and the analysis of social situations. The former refers to the description of a straightforward event or action in such a way as to serve as a persuasive graphic representation of some general normative principle, and the latter signifies the analysis of complex micro-social events to reveal structural characteristics at the macro level (Gluckman, 1955). The extended case technique, in contrast, entails interpreting the connection of structural regularities and the actual unique behavior of people. Another senior sociologist, Van Velsen, also advocated for the use of the case studies beyond their specific context to examine broader social phenomena. He preferred the term “situational analyses” (Van Velsen, 1967, p. 145) where researchers paid attention to the choices individuals made between alternative norms. Furthermore, according to him, the method’s ability to shed light on the intricate connection between a social world of “norms in conflict” and people’s decisions and strategies is what finally makes it worthwhile (Van Velsen, 1967, p. 146). Van Velsen further asserts that scholars may be able to better understand the issue of defining the proper unit of study by extending case studies over a large geographical area.

However, it was their student, Burawoy who further argued that each of these contexts are grounded in the assumption that social phenomena are complex and understanding them requires in-depth exploration (Burawoy, 1998). Burawoy also notes that one of the key features of Extended Case Method is its emphasis on reflexivity. Reflexivity, put simply, entails that researchers are able to reflect on their own positionality and how it shapes their interpretation of the cases that they are studying. Such an outlook helps researchers to avoid reproducing existing power relations and dominant narratives within research. According to Burawoy fostering reflexivity allows researchers to also be more attentive to the nuances and complexities of a phenomenon which is integral to developing new insights. Due to the role of reflexivity, the ECM emerges as a particularly valuable tool for imagining new theoretical insights and refining existing frameworks of research. Within his own career, Burawoy deployed the extended case method in examining the transient nature of race-relations in Zambia. Burawoy took inspiration from Manchester School of Social Anthropology in his career and as a disciple of the school, much of his early work on Zambian copper mines was influenced by what he called “Manchester Marxism” (Burawoy et al., 2000a). Burawoy (1972) drew from the works of Gluckman (1955) for his own treatise on the Zambian Copperbelt. Firstly, he conducted a participant observation based study of Zambian copper industry extended over space and time, then he tried to link the micro-processes he discovered with macro-forces of the colony and the post-colony. Forty years after this study was conducted, Burawoy also conducted an “ethnographic revisit” where he critiqued his own stand-point as a researcher and reconceptualized notions such as “local” and “global” in the wake of the neo-liberal turn. While Burawoy’s contribution led to several studies applying this method to a phenomenon, it must be kept in mind that the execution of such a method maybe time-consuming and resource-intensive. Conducting multiple in-depth case studies and relating it to broader social phenomenon may be a demanding process that is not suitable for researchers with time and resource constraints. Further due to the inherent requirement for in-depth exploration of particular cases, this method maybe limited in its scope for generalizability.

#### 5.1.2 Cluster 2: sociology in the Global South

Based on the co-occurrence of terms such as “Russia”, “Russian economy”; “South Africa” and “South African sociology”, the next theme in Burawoy’s scholarship focuses on topics related to the Global South turn in sociology as well as the Russian and South African labor movements. Burawoy’s interest in Russia is a six-year long project where he, along with his collaborator Krotov, studied the employees of a liquidated furniture enterprise to understand how people survived in the face of unprecedented degeneration of the economy in the late 1990s. In their paper on capitalist modes of production in Russia following the fall of the Soviet Union, Burawoy et al. (2000b) notes that there was a breakdown of the formal economy and workers were forced to adopt one of two survival strategies (1) defensive survival and (2) entrepreneurial survival. In the former, with the loss of jobs and wages, household units sought to rely even more heavily on the traditional Soviet routine of doing business whereas in the latter, experimental ways of doing business and petty commodity production were explored by the workers. This Russian peculiarity has been labelled as a kind of “involution” by Burawoy et al. (2000b) and he further explored the gendered nature of this transition within the socio-economic landscape through several case-studies. He argued that the adoption of these two types of strategies has led to a distinct male dominated pole of wealth generated through the free flow of commodities. Similarly, there has also been a proliferation of a vast underworld of women workers who were forced to subsistence level income based on household industries and kinship networks.

Burawoy’s interest in the Global South was further sustained through his ethnographic project in South Africa, where he examined the interweaving of racist and capitalist behaviors by the colonial state in their treatment of workers in a mining company. In his book *Manufacturing Consent: Changes in The Labor Process Under Monopoly Capitalism* (Burawoy, 1982), Burawoy parsed through the authoritarian policies of a copper mining company in South Africa which appeared to promote black miners in the factory but also avoided any circumstances where a white miner would have to be the subordinate of a black worker and take orders from them. These academic forays into the dynamics of colonial capitalism in the Global South eventually led Burawoy to his study of national regimes of sociology. Specifically, he took note of the Euro-America-centric development of the discipline and the international skew in the division of sociological labor (Burawoy, 2004). Burawoy analysed the pedagogic interventions made within the discipline of sociology in several prominent countries of the Global South like China, Taiwan, Brazil, and Palestine, apart from South Africa, and categorized them into four broad schemes: professional sociology, critical sociology, policy sociology and public sociology. In his later publications, he gave us a clearer picture of what each of these schemes entailed. Burawoy also noted that in the event of such countries contesting Western hegemony in existing scholarship through their polemical writing, the universities of the West often displayed the tendency to subsume such critique by felicitating the critics with laurels and thereby flattening its impact. Eventually, such asymmetries of power, he argued, even percolated into the organization of not just universities but other prominent academic bodies and often hindered the formation of a truly transnational civil society.

#### 5.1.3 Cluster 3: the onset of third wave marketization

Based on co-term analyses in the third cluster, it can be deduced that Burawoy’s industrial sojourns led him to various intriguing settings, from the copper miners of Zambia to the steel plantation workers of Chicago, and he developed that into a class-conscious take-down of the party state and its inability to deliver on their promise of socialism. Terms such as “economic transition”, “market society”, “political practice”, and “marketization” appearing in this cluster indicate his engagement with the turns and twists of capitalist accumulation under neo-liberalism. Most notably in this regard, he engaged with Polanyi (1944) treatise on market society and sought to recalibrate it. Burawoy argued that marketization is not a linear process that follows a singular arc but takes place in waves. The first wave of marketization began in the mid-nineteenth century and resulted in large-scale counter-movements from the working-class which were eventually thwarted in the face of World War I. This period is co-terminus with the advent of political-economy as a concept through the criticism of Marx and Engels. This was followed by the second wave of marketization that took place after the First World War and ended with the state regulation of the 1930s. This period saw the development of Stalinism, Fascism and the New Deal. Finally, Burawoy (2005a) noted that a third and final wave of marketization had begun from the 1970s onwards. Apart from the commodification of money and land, which was somewhat the case for the first wave of marketization as well, there was another big challenge for this wave. The third wave witnessed the commodification and looting of natural resources such as wind, water, air and land which created huge amounts of waste and left large swaths of population in the Global South destitute (Burawoy, 2005a).

In his analyses of the impact of third-wave marketization on regions in the Global South, Burawoy also turned his attention towards India in his piece titled The Future of Sociology (Burawoy, 2007). He signaled that as part of the marketization process, profit-seeking classes in the country collaborated with the regulatory nation-state to produce large-scale commodification of all human-made and natural resources. The only foil to this onslaught, Burawoy adds, is the formation of social movements and the collective will of the public. For the sociologist, this scenario presents four options, they can rely on measures taken by the state to mitigate the harmful effects of the market, as policy sociologists tend to do, or steer clear of such debates as professional sociologists have done. They may also be critical of this marriage between the state and the market. However, according to Burawoy, none of these three choices are advisable since they are ineffective and often incomprehensible. Third-wave marketization in India and the Global South as a whole, characterized by the commodification of labor, money, and land, requires sociologists to seriously think about the discipline’s future and undertake direct public-oriented interventions that can ably support their high-minded critiques of socio-economic crises. This call for expanding the public sphere, especially in the face of rampant exclusion of subaltern groups has been readily taken forward by scholars in India. While most Indian sociologists largely agreed with Burawoy’s observation, some have engaged with his analyses further to highlight that it would be hasty to take these observations and directly apply to the Indian context without modification (Baviskar, 2008). They argue that in countries of the Global South, if the organization of social movements is not led by the local people, they are often anyway perceived as fraudulent by international media and social justice institutions. In such a situation, analytical categories such as the local, the national and the global get enmeshed with one another and are co-constructed (Baviskar, 2008). Nevertheless, public sociology found enormous purchase in India, where governments, state institutions, and other power-holders within the social system frequently fail in their duty to introduce transformative change.

#### 5.1.4 Cluster 4: need for public sociology

The last cluster includes terms such as “multiple publics”, “public sociology”, and “sociological ethos”. It reflects the latest concentration in Burawoy’s scholarship which locates four different types of sociology and especially calls attention to the development of public sociology. It shows that Burawoy, along with his collaborator Laleh Behbahanian, highlighted that public sociology could be developed in three significant ways (1) by identifying and eliminating those forces which hinder the construction of a global civil society, such as global capital or states, (2) by working with the existing global civil societies which are still in their embryonic stage and require further examination, perpetuation and dissemination, and (3) by explicitly and actively partaking in the creation of a global civil society. In that sense, building this global civil society would first require us to explore the basic tenets of public sociology. In his presidential address to the American Sociological Association in 2004 the term public sociology gained prominence and during his long stint as a Professor of Sociology at the University of California, Berkeley, Burawoy, looked at some of the themes that public sociology could explore in detail. At the time of delivering his presidential address, Burawoy’s observations felt like a watershed moment in the development of sociology, which until then was only interested in making cognitive validity claims through normative theory building. He made a call to transgress vocational boundaries and conduct public sociology through the enthusiastic efforts of a deterritorial civil society. According to Burawoy, public sociology is a part of a matrix that includes four different types of sociology, i.e., professional sociology, policy sociology, critical sociology, and finally, public sociology (Burawoy, 2005a).

Professional sociology constitutes multiple formal research programs with intersecting conceptual apparatuses. Critical sociology examines the implicit and explicit foundations of professional sociology, while policy sociology is often conducted in service of a specific goal at the client’s behest. Burawoy mentions that professional sociology, the kind that is practiced within the university space, is concerned with concepts and theorists. In contrast, public sociology is more diverse in that it attempts to bring sociology back to the public from which it emanates and re-engages with it. Burawoy goes on to elucidate the different flavors of public sociology. Firstly, traditional public sociology emerges from the national newspaper columns and opinion pages wherein sociologists write extensively. In this type of public sociology, journalists also play a pivotal role since they carry academic research into the public domain. This, in turn, ignites debate among ordinary people, even though the sociologist might not directly participate in it. Another way of practicing sociology, which Burawoy has called organic public sociology, brings the two strands of academic and non-academic into conversation with one another. This usually happens when sociologists work closely with a dense, organized counter-public. Thus, public sociology is perhaps Burawoy’s most comprehensive attempt at reshaping the theory and practice of sociology. In the decade since the arrival of public sociology in the disciplinary mainstream, following Burawoy’s call-to-action, a surge of research has attempted to take sociological knowledge to the non-academic audience. Particularly, in the Global South, with its historical legacy of exclusions, there is a need to reflect seriously on the utility of public sociology without getting caught up in the typology. It is therefore crucial to point out that even though the premise of Burawoy’s concept hinges upon a distinction between professional, and public sociology, it may be argued that such water-tight compartmentalization are untenable, given the possibility that a single sociologist may occupy more than one quadrant at a single point or at various junctures in their career (Ericson, 2005). Thus, all sociology, weather explicitly public or not, should generate a degree of civic engagement. Even as research and teaching become a lucrative, for-profit initiative and there is an ever-increasing influence of money power and political muscle, young scholars of the discipline must ask themselves how sociology can be helpful to the public and what measures of course correction can be taken to reduce public indifference towards the discipline.

Through thematic mapping and synthesis of all four clusters, it is possible to reveal the research foci, locate scopes of interest and relate it to corresponding institutional contexts.

### 5.2 Temporal analyses

**Figure 4:**
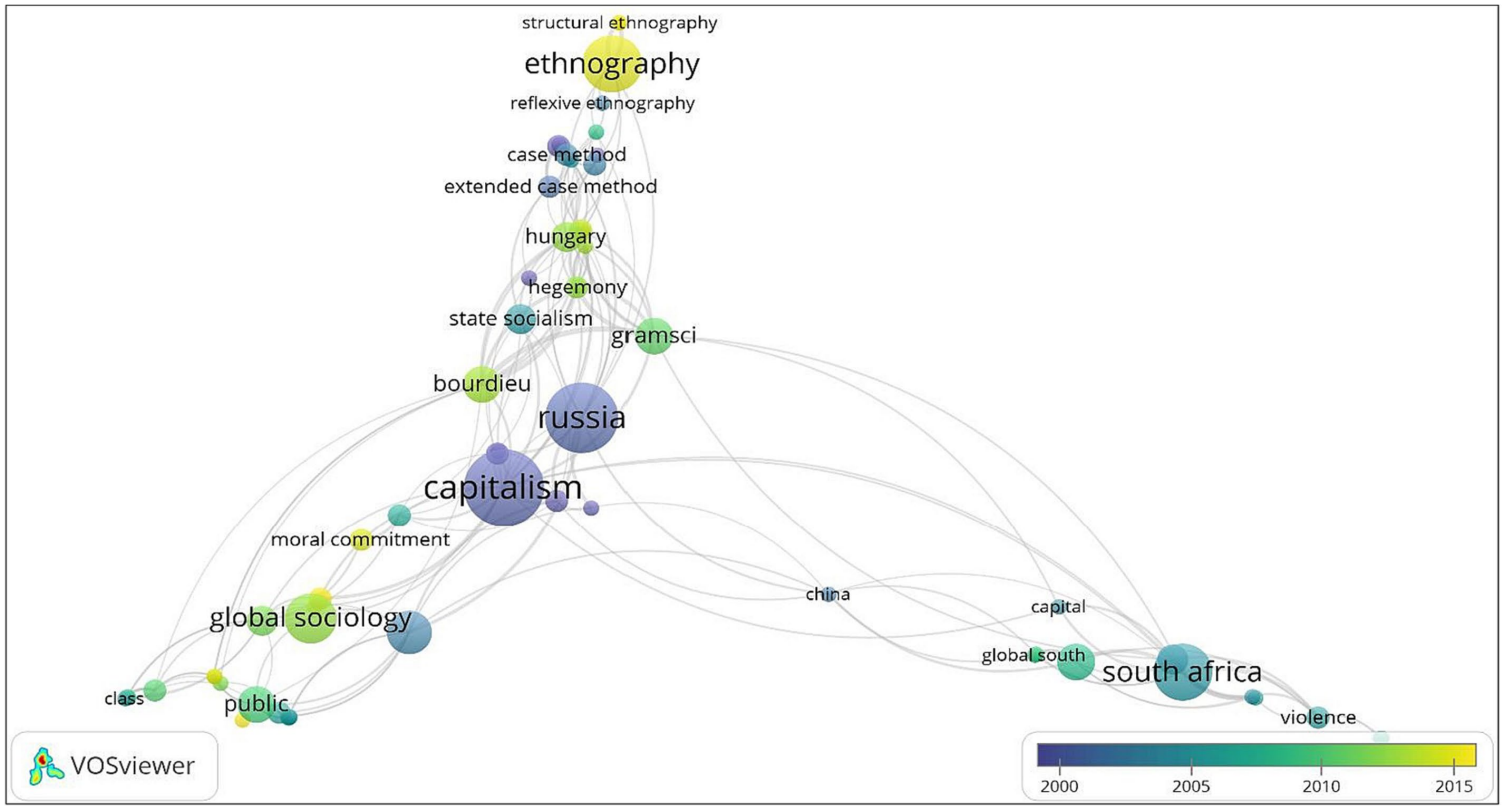
Temporal visualization of Burawoy’s scholarship based on term co-occurrence and average publications per year. Source: Generated by authors from VOSviewer.

Temporal analyses of Burawoy’s scholarship was done through overlay visualization on VOSviewer. This map is identical to the network visualization map except that this corresponding functionality uses both term co-occurrence linkages as well as average publication per year scores to interpret the year to year evolution of research topics covered in the retrieved literature within the given time period. Two shades of colors, dark purple and yellow are used by the software on a continuum to represent time-variance in key-word co-occurrences where the former signifies topics covered earlier and the latter represents topics studied more recently. In Figure 4, the timescale represents the years between 2000 and 2015 because the visualization is based on the average publication year of the articles in which a specific term appeared. We can interpret from the figure that there has been a shift in the main focus of research for Burawoy. His early research, represented by the dark purple region that is on the left side of the timescale, tended to explore labor relations and how that in turn is designed to control and dominate industrial workers in different socio-political contexts such as U.S.A and Africa. However, from the 2000s onwards, his scholarship on public sociology and the theorization on extended case method has taken on an increasingly central role. This is represented by the yellow section on the right side of the timescale. These emergent trends in research provides a more robust understanding of the shifts in Burawoy’s long scholarly oeuvre and encourages other scholars to carve out a niche in sociological knowledge that is about the public and for the public.

## 6 Conclusion

This paper enabled us to argue that bibliometric analyses can be an effective complement to traditional review methods through qualitative synthesis and aides researchers to gain in-depth insights about the academic research output of not just Burawoy but any sociologist. Too often, sociology is entangled in lofty theorization that merely reproduces patterns of thought dominant in the West without orienting itself adequately to local contexts. A kernel of this argument can be found throughout Burawoy’s own academic research career, be it in the topics he chose to work on or the entities he chose to collaborate with. While the standing of Michael Burawoy as a scholar can always be effectively gauged through detailed engagement with his scholarship, bibliometric inquiry helps to consolidate the fragmented structure of knowledge production within sociology. Burawoy has himself noted that digital media has set a new standard for how local pedagogy and its dialogue with the global takes place (Behbehanian and Burawoy, 2014). However, it should be noted that the present analyses comes with a limitation. Since this paper focused on Burawoy’s publications within the field of sociology, the generalizability of the findings are relative to the growth of knowledge within the discipline. Therefore, one important future direction of work would be to link the type of bibliometric indicators analysed here to data from Burawoy’s publications in other disciplines such as history (Burawoy, 1990) and labor studies (Burawoy, 2010). The analyses in this paper provides a strong empirical base and some cursory guidelines on which later scholarship can be built to assess the contribution of Burawoy, or other sociologists, in other interdisciplinary areas. There are two major reasons to argue for the use of the bibliometric method for studying the work of other theorists: coverage and collaboration. Firstly, bibliometric analyses provides ready access to aggregated data from diverse research journals and therefore provides in-depth coverage of literature which is important to gauge future research directions, secondly, it can track collaborations that a researcher makes with various institutions and other researchers, which this paper argues is integral to the building of a cohesive academic community.

## Author contributions

AR: Conceptualization, Supervision, Writing – original draft, Writing – review & editing. AS: Data curation, Formal analysis, Writing – original draft, Writing – review & editing.

## References

[ref1] Baviskar A. (2008). Pedagogy, public sociology and politics in India: what is to be done? Curr. Sociol. 56, 425–433. doi: 10.1177/0011392107088236

[ref2] Behbehanian L. Burawoy M. (2012). A pedagogy for the global. Contexts 11, 80–83. doi: 10.1177/1536504212436510

[ref3] Behbehanian L. Burawoy M. (2014). Appendix: global pedagogy in a digital age. Curr. Sociol. 62, 285–291. doi: 10.1177/0011392113515799

[ref4] Bridger J. C. Alter T. R. (2010). Public sociology, public scholarship, and community development. Community Dev. 41, 405–416. doi: 10.1080/15575330.2010.519039

[ref5] Burawoy M. (1972). The colour of class on the Copperbelt: from African advancement to Zambianization. Manchester: Manchester University Press.

[ref6] Burawoy M. (1982) Manufacturing consent: changes in the labour process under monopoly capitalism. Chicago: University of Chicago Press.

[ref7] Burawoy M. (1990). Marxism as science: historical challenges and theoretical growth. Am. Sociol. Rev. 55:775. doi: 10.2307/2095745

[ref8] Burawoy M. (1998). The extended case method. Sociol Theory 16, 4–33. doi: 10.1111/0735-2751.00040

[ref9] Burawoy M. (2004). Public sociologies: contradictions, dilemmas, and possibilities. Soc. Forces 82, 1603–1618. doi: 10.1353/sof.2004.0064

[ref10] Burawoy M. (2005a). For public sociology. Am. Sociol. Rev. 70, 4–28. doi: 10.1177/000312240507000102 15926908

[ref11] Burawoy M. (2005b). Response: public sociology: populist fad or path to renewal? Br. J. Sociol. 56, 417–432. doi: 10.1111/j.1468-4446.2005.00075.x 15926908

[ref12] Burawoy M. (2007). The future of sociology. Sociol. Bull. 56, 83–98. doi: 10.1177/0038022920070301

[ref14] Burawoy M. (2010). From Polanyi to Pollyanna: the false optimism of global labor studies. Glob. Labour J. 1, 304–307. doi: 10.15173/glj.v1i2.1079

[ref15] Burawoy M. (2013). “Public sociology: the task and the promise” in Ten lessons in introductory sociology. eds. Gould K. Lewis T. (Oxford University Press), 278–299.

[ref16] Burawoy M. Krotov P. (1992). The soviet transition from socialism to capitalism: worker control and economic bargaining in the wood industry. Am. Sociol. Rev. 57, 16–38. doi: 10.2307/2096142

[ref17] Burawoy M. Krotov P. Lytkina T. (2000b). Involution and destitution in capitalist Russia. Ethnography 1, 43–65. doi: 10.1177/14661380022230633

[ref18] Burawoy M. Blum A. J. George S. Gille Z. Thayer M. Gowan T.. (2000a). Global ethnography: forces, connections, and imaginations in a postmodern world. Berkeley, CA: University of California Press.

[ref19] Dong X. Wei X. Shu F. Su Q. Wang J. Liu N.. (2022). A bibliometric analysis on global psychological and behavioural research landscape on COVID-19 pandemic. Int. J. Environ. Res. Public Health 19:879. doi: 10.3390/ijerph19020879 35055700 PMC8776113

[ref20] Ericson R. (2005). Publicizing sociology. Br. J. Sociol. 56, 365–372. doi: 10.1111/j.1468-4446.2005.00066.x 16156747

[ref21] Evans-Pritchard E. E. (1937). Witchcraft, oracles and magic among the Azande. London: Oxford University Press.

[ref22] Evens T. M. S. Handelman D. (2005). Preface: historicizing the extended-case method. Soc. Anal. 49, 123–128. doi: 10.3167/015597705780274986

[ref23] Fortes M. (1949). The web of kinship among the Tallensi: the second part of an analysis of the social structure of a trans-Volta tribe. London: Oxford University Press.

[ref25] Gluckman M. (1955). Anthropology in Central Africa. J. R. Soc. Arts 103. Available at: https://www.jstor.org/stable/41364719

[ref26] Holmwood J. (2007). Sociology as public discourse and professional practice: a critique of Michael Burawoy. Sociol Theory 25, 46–66. doi: 10.1111/j.1467-9558.2007.00297.x

[ref27] Kempny M. (2005). History of the Manchester “School” and the extended-case method. Soc. Anal. 49, 144–165. doi: 10.3167/015597705780275057

[ref28] Korom P. (2020). The prestige elite in sociology: toward a collective biography of the most cited scholars (1970–2010). Sociol. Q. 61, 128–163. doi: 10.1080/00380253.2019.1581037, PMID: 32256226 PMC7077350

[ref29] Pathak D. N. (2020). Public sociology in South Asia during lockdown. Glob. Dialogue 10, 25–26.

[ref30] Pathak D. N. (2022). Being and becoming (of/with) Burawoy: an anxious apprehension of public sociology in South Asia. Int. Sociol. Rev. 37, 557–568. doi: 10.1177/02685809221138018

[ref31] Petrovich E. (2022). Bibliometrics in press: representations and uses of bibliometric indicators in the Italian daily newspapers. Scientometrics 127, 2195–2233. doi: 10.1007/s11192-022-04341-6

[ref32] Polanyi K. (1944). The great transformation: economic and political origins of our time. New York: Rinehart.

[ref33] Pranckute R. (2021). Web of science (WoS) and Scopus: the titans of bibliographic information in today’s academic world. Publica 9:12. doi: 10.3390/publications9010012

[ref34] Pritchard A. Wittig G. R. (1981). Bibliometrics. Watford: AIIM Books.

[ref35] Samudra A. (2023). Entrepreneurship and digital economy – a bibliometric analysis. SDMIMD J. Manag. 14, 9–24. doi: 10.18311/sdmimd/2023/32449

[ref36] Sun Y. Wang G. Feng H. (2021). Lingu istic studies on social media: a bibliometric analysis. SAGE Open 11:215824402110475. doi: 10.1177/21582440211047572

[ref37] Sztompka P. Burawoy M. (2011). Debate on international sociology. Contemp. Sociol. 40, 388–396. doi: 10.1177/0094306111412512

[ref38] Turner V. Abrahams R. Harris A. (2017). The ritual process: structure and anti-structure. London: Routledge.

[ref39] Van Velsen J. (1967). “The extended case method and situational analysis” in The craft of social anthropology. ed. Epstein A. L. (London: Routledge), 129–149.

